# A meta‐analysis of category fluency clustering and switching abilities in mild cognitive impairment and Alzheimer's disease

**DOI:** 10.1002/alz70857_105557

**Published:** 2025-12-25

**Authors:** Matteo De Marco, Sonia Di Tella, Laura M Wright

**Affiliations:** ^1^ Brunel University London, Uxbridge, Middlesex, United Kingdom; ^2^ Catholic University of the Sacred Heart, Milan, Milan, Italy; ^3^ Translational and Clinical Research Institute, Newcastle University, Newcastle upon Tyne, United Kingdom

## Abstract

**Background:**

We ran a meta‐analysis to characterise clustering and switching abilities of adults with mild cognitive impairment and Alzheimer's dementia (MCI/AD), during their Category Fluency (CF) performance.

**Method:**

A bibliographical search was carried out in *Web of Knowledge*, *MEDLINE*, *CINAHL Plus*, *APA PsycArticles*, *APA PsycINFO* and *Academic Search Complete* databases, via Web of Science, Pubmed, Ebsco Host and Ovid. The search string included the terms “*fluency*”, “cluster*” and a third one taken from a set of alternatives defining the conditions of interest (i.e., MCI/AD). A total of 428 unique entries were shortlisted. After screening and eligibility‐checking procedures, 29 studies were retained for inclusion. Mean cluster size (MCS) and number of switches (NOS) were extracted as outcomes. Random‐effect meta‐analysis models were run using ProMeta3, to compare MCI/AD individuals and cognitively‐healthy controls. Age, education, Mini Mental State Examination‐equivalent (MMSE) scores, category type and standard CF counts were included as moderators.

**Result:**

The meta‐analysis of MCS included 2,066 controls and 1,672 MCI/AD individuals, and revealed that this latter cohort generated significantly smaller clusters (*G* = 0.30, *p* < 0.001). No publication bias was found (Egger's test's: *p* = 0.068), but the analyses were influenced by significant study‐to‐study heterogeneity (*Q* = 82.14, *p* < 0.001). No moderator analysis reached statistical significance. The meta‐analysis of NOS included 3,632 controls and 1,778 MCI/AD individuals, and revealed that this latter cohort made significantly fewer switches (*G* = 0.85, *p* < 0.001). Further analyses indicated no evidence of publication bias (Egger's test's: *p* = 0.737), but significant study‐to‐study heterogeneity (*Q* = 203.34, *p* < 0.001). Standard CF counts and MMSE scores were reported as significant moderators, both as negative predictors of effect size.

**Conclusion:**

CF MCS and NOS are significantly reduced among individuals with MCI/AD. Clustering is thought to be driven by automatic semantic processing, while switching is considered the controlled component of CF. People who generate larger clusters are able to retrieve more exemplars of a subcategory, while those who make more switches can identify (and “exploit”) more thematic directions during the test. This highlights the role of both semantic knowledge and semantic control in CF decline in individuals with MCI/AD. Quantifying MCS and NOS can support the assessment of semantic processing when CF is administered.

